# Comparative study of modified midline lumbar interbody fusion and modified transforaminal lumbar interbody fusion for the treatment of single-level lumbar degenerative diseases

**DOI:** 10.1186/s12893-025-03100-7

**Published:** 2025-08-22

**Authors:** Yuanpeng Yue, Yihui Liu, Ce Dong, Zhenyu Wang

**Affiliations:** Department of Orthopedics, Spine Surgery, The First Hospital of Jilin University, Jilin University, 1 Xinmin Street, Changchun, 130000 China

**Keywords:** Cortical bone trajectory screw, Midline lumbar interbody fusion, Pedicle screw, Transarticular surface screw, Transforaminal lumbar interbody fusion

## Abstract

**Purpose:**

To evaluate the safety and efficacy of modified cortical bone trajectory (MCBT) screw combined with transarticular screw (TASS) fixation (MCBT-TASS) in modified midline lumbar interbody fusion (M-MIDLIF) for single-level lumbar degenerative disease (LDD).

**Methods:**

We retrospectively included 104 patients with L4–5 or L5–S1 single-segment LDD who had indications for decompression, fusion, and internal fixation surgery from 2019 to 2022. They were subsequently divided into M-MIDLIF and modified transforaminal lumbar interbody fusion (M-TLIF) groups according to the surgical approach. Basic demographic, surgical, and radiological data, as well as clinical outcomes (Oswestry Disability Index (ODI) and visual analog scale (VAS) scores), were collected.

**Results:**

Basic demographic data, fusion rates, postoperative hospital stays, and follow-up times did not significantly differ between the two groups. Compared with those in the M-TLIF group, the intraoperative blood loss (68.57 ± 14.84 mL) and postoperative drainage volume (33.93 ± 9.17 mL) in the M-MIDLIF group were lower (intraoperative blood loss: 171.79 ± 12.78 mL, *p* < 0.05; postoperative drainage volume: 65.36 ± 10.36, *p* < 0.05). In the M-MIDLIF group, there was no significant difference in the radiographic recognizable rate (91.07%) or intraoperative visual recognizable rate (87.50%) of internal inverted chevron-shaped (V-shaped) crests. The optimal position screw rates for the MCBT, TASS, and traditional pedicle screw (TPS) methods were 94.64%, 94.64%, and 87.5%, respectively, and the differences were not significant. MCBT technology preserved the integrity of the posterior ligamentous complex (PLC) in 92.85% of patients in the M-MIDLIF group. The facet joint violation (FJV) rate of MCBT screws (3.57%) was lower than that of TPS screws (14.29%). Compared with the M-TLIF group, the M-MIDLIF group presented greater reductions in the ODI and VAS scores for both low back and leg pain at 1 week postoperatively (*P* < 0.05). However, no statistically significant differences in these scores were observed between the two groups at later time points (*p* > 0.05).

**Conclusion:**

M-MIDLIF can achieve decompression, fixation, and fusion via a median incision while preserving the integrity of the posterior ligamentous complex. In the treatment of single-level lumbar degenerative disease, M-MIDLIF has comparatively enhanced minimally invasive advantages over M-TLIF during the perioperative period while maintaining non-inferior clinical safety and efficacy relative to M-TLIF.

## Introduction

Transforaminal lumbar interbody fusion (TLIF) is a minimally invasive lumbar spine surgical technique that involves a bilateral incision through the Wiltse approach, thereby preserving the integrity of the posterior ligamentous complex (PLC) [1–4]. Facet joint resection, decompression, TPS fixation, and fusion should be completed via bilateral incisions. Therefore, this study proposed that preserving partial facet joints in M-TLIF and combining them with facet joint fusion can maintain the lumbar spine’s postoperative stability [5–9]. Inserting a TPS in osteoporotic patients may carry the risk of screw loosening within the body. Therefore, in 2009, Santoni proposed a cortical bone trajectory (CBT) screw technique to increase the contact area and pullout strength of screws with cortical bone, thereby increasing its effectiveness for minimally invasive lumbar spine surgery [10–12]. Through a posterior midline incision, midline lumbar interbody fusion (MIDLIF) enables simultaneous decompression, CBT screw fixation, and fusion, representing a minimally invasive surgical technique. CBT screws not only lack easily identifiable and reproducible anatomical landmarks but also often require destruction of the PLC to meet placement standards [13–16]. Preserving the integrity of the PLC contributes to the postoperative stability of the lumbar spine and reduces postoperative complications [6, 17]. Therefore, this study aimed to propose the internal inverted V-shaped crest as an easily identifiable and reproducible anatomical reference landmark for guiding MCBT screw placement to preserve the integrity of the PLC. The feasibility of applying TASS to the S1 vertebra has been anatomically confirmed [18]. Prior studies demonstrated that CBT-TASS fixation in PLIF resulted in superior perioperative outcomes compared to traditional pedicle screws via the Wiltse approach but presented certain limitations: (1) extensive resection of posterior elements (facets, lamina, and PLC), compromising spinal stability; (2) CBT screw insertion at the pedicle stump surface, reducing entry-point stability and increasing the risk of screw loosening and pedicle breach; and (3) TASS application limited to the S1 level [19]. This study introduces the reproducible inverted V-shaped crest as an anatomical landmark for MCBT screw insertion to preserve the integrity of the PLC and provides preliminary clinical evidence supporting its application in lumbar TASS. It is currently unclear whether the M-MIDLIF procedure achieves good clinical outcomes by performing decompression, MCBT screws combined with TASS fixation, and fusion within a posterior midline incision to preserve the PLC and minimize surgical complications.

## Materials and methods

### Patients

This study was approved by the Ethics Committee of the First Bethune Hospital of Jilin University. We included 104 patients with single-segment lumbar degenerative disease, including lumbar disc herniation and lumbar spinal stenosis, at L4-5 or L5-S1 who underwent lumbar decompression, diskectomy, bone graft fusion, and internal fixation from 2019 to 2022. Fifty-three patients underwent modified bilateral transforaminal lumbar interbody fusion (M-TLIF) via the Wiltse approach and were categorized into the M-TLIF group, whereas 51 patients who underwent modified midline lumbar interbody fusion (M-MIDLIF) were designated into the M-MIDLIF group. The inclusion criteria were patients aged 18–70 years; those with single-segment LDD at L4–5 or L5–S1; those with indications for fusion and fixation surgery; and patients with complete follow-up records or imaging data. The exclusion criteria were patients with incomplete follow-up records or imaging data; those with a history of previous lumbar spine surgery; and patients with congenital deformities, tumors, infections, or lumbar spine trauma.

### Operative method

#### M-MIDLIF

**Fig. 1:**
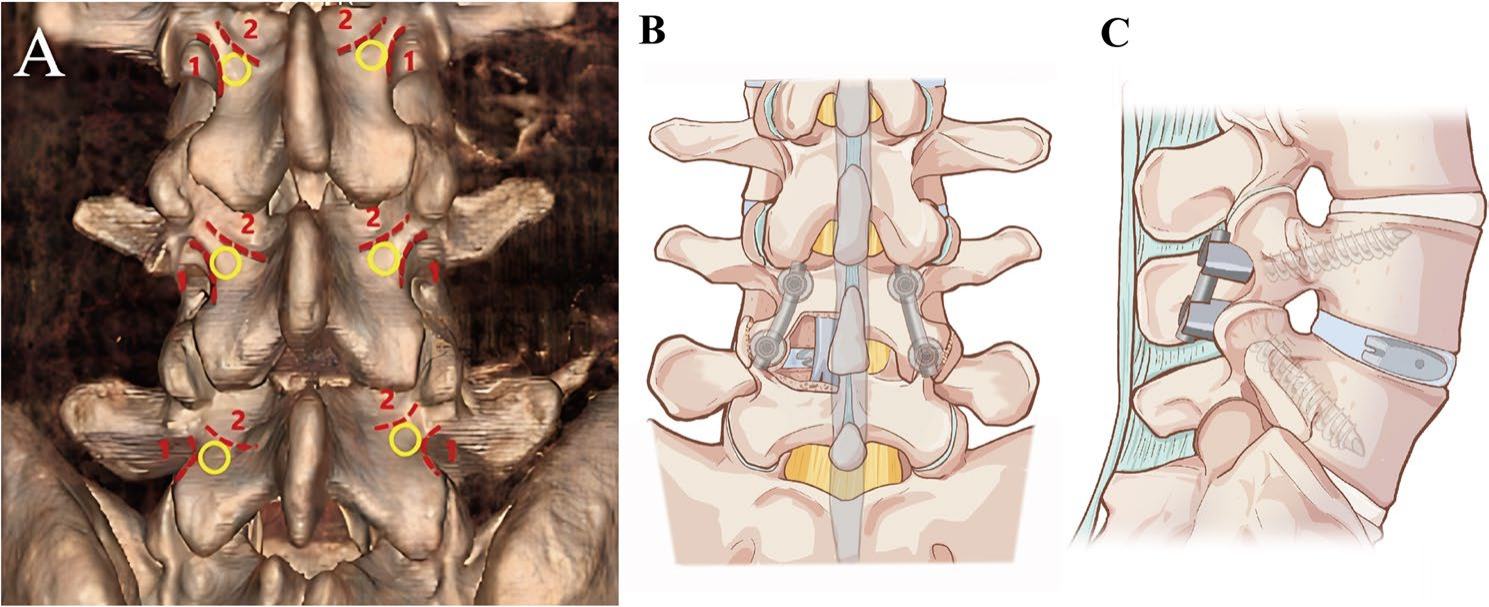
**A** Curve 1 represents the outer crest of the internal inverted V-shaped crest, and Curve 2 represents the inner crest of the internal inverted V-shaped crest. The internal inverted V-shaped crest was composed of the outer and inner crests of the internal inverted V-shaped crest. The MCBT screw was accessed at the yellow circular area with a radius of about 3.0 mm, tangentially to curves 1 and 2. **B** and **C** Illustrations demonstrating a complete M-MIDLIF construct

A midline incision was made 2–4 cm superior to the spinous process of the inferior vertebral body. The incision was deepened through the skin, subcutaneous tissue, and lumbodorsal fascia to expose the spinous process. The spinous process was dissected along the vertebral lamina, and the periosteum was stripped from below and peeled outward to the inner edge of the articular process. After the isthmus’ outer edge was visualized, it was stripped upward to the area near the top of the internal inverted V-shaped crest, exposing the entire upper vertebral lamina. Furthermore, it traversed downward to 1/3 of the upper part of the lower vertebral lamina. The inferior half and the superior one-third of the superior and inferior vertebral laminae were removed, respectively. We also separated the inner 2/3 of the inferior articular process from the upper vertebral body and the inner half of the superior articular process of the lower vertebral body. Moreover, 1/4 of the lower articular process of the upper vertebral body on the non-decompression side was detached, thereby exposing the upper articular surface of the lower vertebral body. The hypothetical circle with an about 3.0 mm radius was tangential to the inner and outer crests of the internal inverted V-shaped crest; the circle’s center was designated the MCBT screw insertion point (Fig. 1A). Before surgery, the surgeon reconstructs the patient’s lumbar 3D CT image to identify the anatomical location of the internal inverted V-shaped crest and measures the width and height of the patient’s pedicle, as well as the screw length and angle. After confirming the anatomical landmarks of the internal inverted V-shaped crest during surgery, the appropriate MCBT screws are inserted.

TASS was inserted into the S1 vertebral body following Terai’s surgical procedure [19]. The entry point on the decompression side of the L4 and L5 vertebral bodies is located about at the inferior third of the vertical midline of the articular surface of the superior articular process. On the non-decompression side, the entry point for the TAS screw in the L4 and L5 vertebrae is positioned in the outer lower quarter of the articular surface of the superior articular process. Moreover, in the L5 vertebra, tilting for TASS should be < 10° to the vertical direction in the axial plane, whereas the caudal angle should be tilted about 10–30° in the sagittal plane. After the MCBT screws and TASS were placed in the direction of the screw pathway at the insertion point, the MCBT screw trajectory was similar to that of the ideal CBT screw [14].

Additionally, a suitably sized PEEK cage was implanted according to the intraoperative cage trial model, followed by intervertebral bone grafting. We also completely removed the surface cartilage and non-decompression side articular process cortical bone and completed graft bone fusion between the articular processes (Fig. 1B, C).

#### M-TLIF

**Fig. 2:**
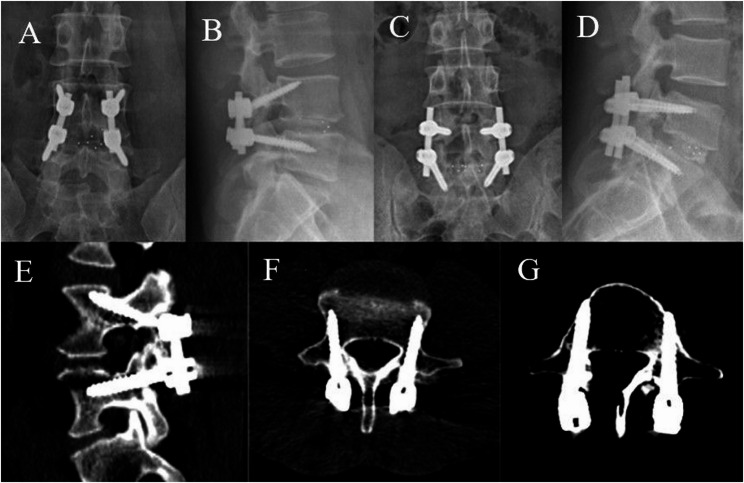
Anteroposterior and lateral radiographs after MCBT screw and TASS fixation (**A**, **B**) and pedicle screw fixation (**C**, **D**). **E** and **F**, Axial and sagittal computed tomography images of the L4 cortical bone trajectory. **E** and **G**, Axial and sagittal computed tomography images of the L5 TASS trajectory

A paramedian incision 2 to 3 cm lateral to the midline was made through the lumbar dorsal fascia via the bilateral Wiltse approach. The incision was about 2–3.5 cm in length and longitudinally downward between the multifidus and longissimus muscles. TPS were inserted at the top of the inverted V-shaped crest or at the junction of the transverse process and the outer edge of the superior articular process [20]. The extent of resection of the facet joint and lamina, as well as the extent of interbody and facet joint fusion on the non-decompression side, was the same as that in the M-MIDLIF group. Additionally, TPS fixation was performed before or after the M-TLIF procedure (Fig. 2C, D).

### Clinical outcome evaluations

We examined the visual analog scale (VAS) score and Oswestry Disability Index (ODI) preoperatively and at 1 week, 3 months, 1 year, and 2 years postoperatively to determine the clinical outcomes. We also retrospectively reviewed and analyzed surgical time, intraoperative bleeding, incision length, and surgical complications.

**Fig. 3:**
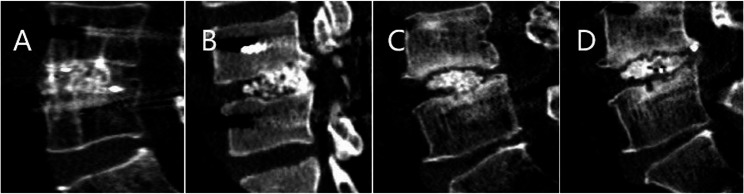
The Bridwell grading system is composed of the following categories: **A** fusion with remodeling and the presence of trabeculae (Grade I); **B** intact graft but not fully remodeled and incorporated as well as absence of lucency (Grade II); **C** intact graft with potential lucency at the top and bottom of the graft (Grade III); **D** absence of fusion with collapse/resorption of the graft (Grade IV)

**Fig. 4:**
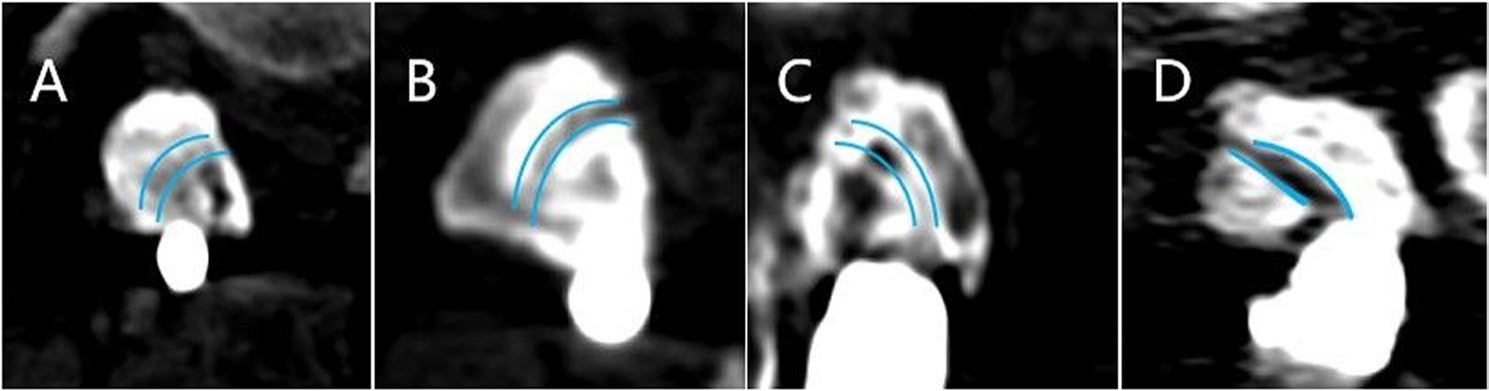
The blue curve represents the preoperative facet articular surface. **A** Grade I was defined as complete bony continuity covering the entire facet joint. **B** Grade II was designated partial bony continuity on a facet joint. **C** In Grade III, bony continuity was not confirmed at any point along a facet joint; **D** Grade IV was defined as an obvious nonunion of a facet fusion. While unilateral or bilateral grade I or II fusions were considered adequate, grade III or IV fusions were designated inadequate

### Radiological evaluation

We collected the imaging data using the Philips Brilliance 64 CT system for scanning, with a slice thickness and interval of 1 mm. Subsequently, the sagittal, coronal, and axial views of the lumbar spine CT were reconstructed in the Neusoft PACS/RIS workstation (version 5.5.0.20053), achieving length and angle accuracies of 0.1 mm and 0.1°, respectively.

The screw placement accuracy, screw angle, and assessment of facet joint invasion, as well as interbody and facet fusion, were evaluated blindly by two independent researchers via postoperative CT scans. Interbody fusion and facet fusion assessments were conducted at the final follow-up time point to allow sufficient time for bony fusion to occur. Any discrepancies in classification or grading were subsequently resolved by a third independent observer. Furthermore, the most unfavorable circumstances were also reflected when the critical positions were automatically downgraded.

Based on postoperative CT scans, PS, MCBT screw, and TASS were evaluated for accuracy within the vertebral pedicle using the Gertzbein-Robbins scale [21]. The Gertzbein-Robbins scale is classified into grade A (screw position completely within the pedicle), grade B (cortical breach of < 2 mm), grade C (cortical breach of ≥ 2 mm but < 4 mm), grade D (cortical breach of ≥ 4 mm but < 6 mm), and grade E (cortical breach of ≥ 6 mm). Thus, Grade A was regarded as the optimal screw position. While grades A and B indicated clinically acceptable screw positions, grades C, D, and E were considered misplaced placements. The Babu grading system was used to assess proximal facet joint violation [22]. Grade 0 referred to a screw that was outside the facet and did not encroach on the facet joint. If the screw was in the lateral facet but did not infringe on the articular facet, it was labeled Grade 1. Grade 2 referred to the screws that penetrated the articular facet by ≤ 1 mm, while the screws within the facet’s articular surface were labeled Grade 3. All screws were graded by evaluating axial images with the coronal and sagittal reconstructed representations. The Bridwell interbody fusion grading system was used to assess interbody fusion [23] (Fig. 3). Grading system for the facet fusion assessment on an axial computed tomography image [7] (Fig. 4).

### Statistical analysis

IBM SPSS Statistics version 27.0 software was used for the statistical analysis. To address potential selection bias inherent in the non-randomized retrospective design, propensity score matching (PSM) was performed. Propensity scores were generated via a logistic regression model with the surgical approach as the dependent variable and the following covariates: age, body mass index (BMI), surgical segment, and sex. Patients were matched 1:1 via nearest-neighbor matching with a caliper width of 0.02 standard deviations of the logit propensity score. All subsequent analyses were conducted on the matched cohort. The weighted kappa coefficient was calculated to assess inter-observer agreement for ordinal data. The normally distributed variables were evaluated by the Shapiro-Wilk normality test, and the continuous variables are presented as the means and standard deviations. Independent or paired sample t-tests were used to compare differences between two groups for normally distributed data. For non-normal data, we used the Mann-Whitney U test. ANOVA was performed within each group to compare the results before and after treatment. The categorical variables are expressed as absolute (no.) and relative (%) frequencies, followed by the application of the chi-square test. Fisher’s exact test was used when the sample size was < 40 or the expected or theoretical frequency in any cells was < 1. A *p*-value < 0.05 was considered to indicate statistical significance.

## Results

### Patient demographics

This study included 104 patients who underwent treatment using the M-MIDLIF (51 cases) and the M-TLIF (53 cases) methods. Before surgery, no statistically significant differences were observed in demographic characteristics, surgical segment, or follow-up time (Table 1). After propensity score matching, 28 pairs of patients were successfully matched. No significant differences in age, BMI, surgical segment, or sex remained between the M-MIDLIF and M-TLIF groups (all SMD < 0.1; Table 1).

**Table 1:** Descriptive statistics of the study patients

Variables	M-MIDLIF (*n* = 51)	M-TLIF (*n* = 53)	t/Z/X^2^	*p* value
Unadjusted				
Age (years)	45.94 ±11.76	46.38 ±13.79	0.173	0.863
Gender (male/female)	27/24	28/25	0.000	1.000
BMI (kg/m^2^)	24.42 ±5.08	24.33 ±4.12	−0.410	0.369^#^
Follow-up time (month)	23.47 ±3.78	23.30 ±4.44	−0.483	0.629^#^
Level of screw instruments				
L4-5	17	31	6.619	0.10
L5-S1	34	22	6.619	0.10
Propensity Matched	M-MIDLIF (*n* = 28)	M-TLIF (*n* = 28)		
Age (years)	46.68 ± 14.84	50.50 ± 10.88	−1.451	0.152
Gender (male/female)	12/16	16/12	1.43	0.285
BMI (kg/m^2^)	23.96 ± 6.03	23.39 ± 2.57	−1.386	0.166
Level of screw instruments			0.072	0.789
L4-5	13	14		
L5-S1	15	14		

^#^ *p*-value was calculated by the Mann-Whitney U test

### Perioperative and postoperative metrics

Compared with the M-TLIF group, the M-MIDLIF group demonstrated a 60.08% reduction in intraoperative blood loss (*p* < 0.001) and a 48.09% decrease in postoperative drainage volume (*p* < 0.001). The surgical time and complications, postoperative hospital stay, and time to get out of bed after surgery did not differ significantly between the two groups (*p* > 0.05). MCBT technology preserved the integrity of the posterior ligament complex in 92.85% of patients in the M-MIDLIF group. In the M-MIDLIF group, there was no significant difference in the radiographic recognizable rate (91.07%) or intraoperative visual recognizable rate (87.50%) of the internal inverted V-shaped crest (Table 2).

**Table 2:** Summary of surgical outcomes between M-MIDLIF and M-TLIF groups

Variables	M-MIDLIF (*n* = 28)	M-TLIF (*n* = 28)	t/Z/X^2^	*p* value
Surgical time from skin to skin (h)	4.10 ± 0.92	4.18 ± 0.81	−0.352	0.726
Intraoperative blood loss (ml)	68.57 ±14.84	171.79 ±12.78	−6.492	0.001<^#^
Postoperative drainage (ml)	33.93 ±9.17	65.36 ±10.36	−6.29	0.001<^#^
Surgical incision length(cm)	3.42 ±0.79	L:3.11 ±0.54 R:2.89 ±0.62		
Integrity of the PLC	26(92.85%)	28(100%)		0.491*
Surgical Complications				
Dural tear	1(3.57%)	1(3.57%)		1.000*
Cage retropulsion and subsidence	1(3.57%)	2(7.14%)		1.000*
Recognizable rate of internal “˄” shape crest			0.373	0.541
Radiographic recognizable rate	51(91.07%)	-		
Intraoperative visual recognizable rate	49(87.50%)	-		
Postoperative hospital stay(Day)	9.39 ± 3.93	9.00 ± 2.86	−0.207	0.836
Time to get out of bed after surgery (Day)	5.71 ± 1.10	5.75 ± 1.10	−0.138	0.890

*L* Left and *R* Right

*p- value was calculated by Fisher’s precision probability test

^#^ *p*-value was calculated by the Mann-Whitney U test

### Comparison of clinical outcomes

**Fig. 5:**
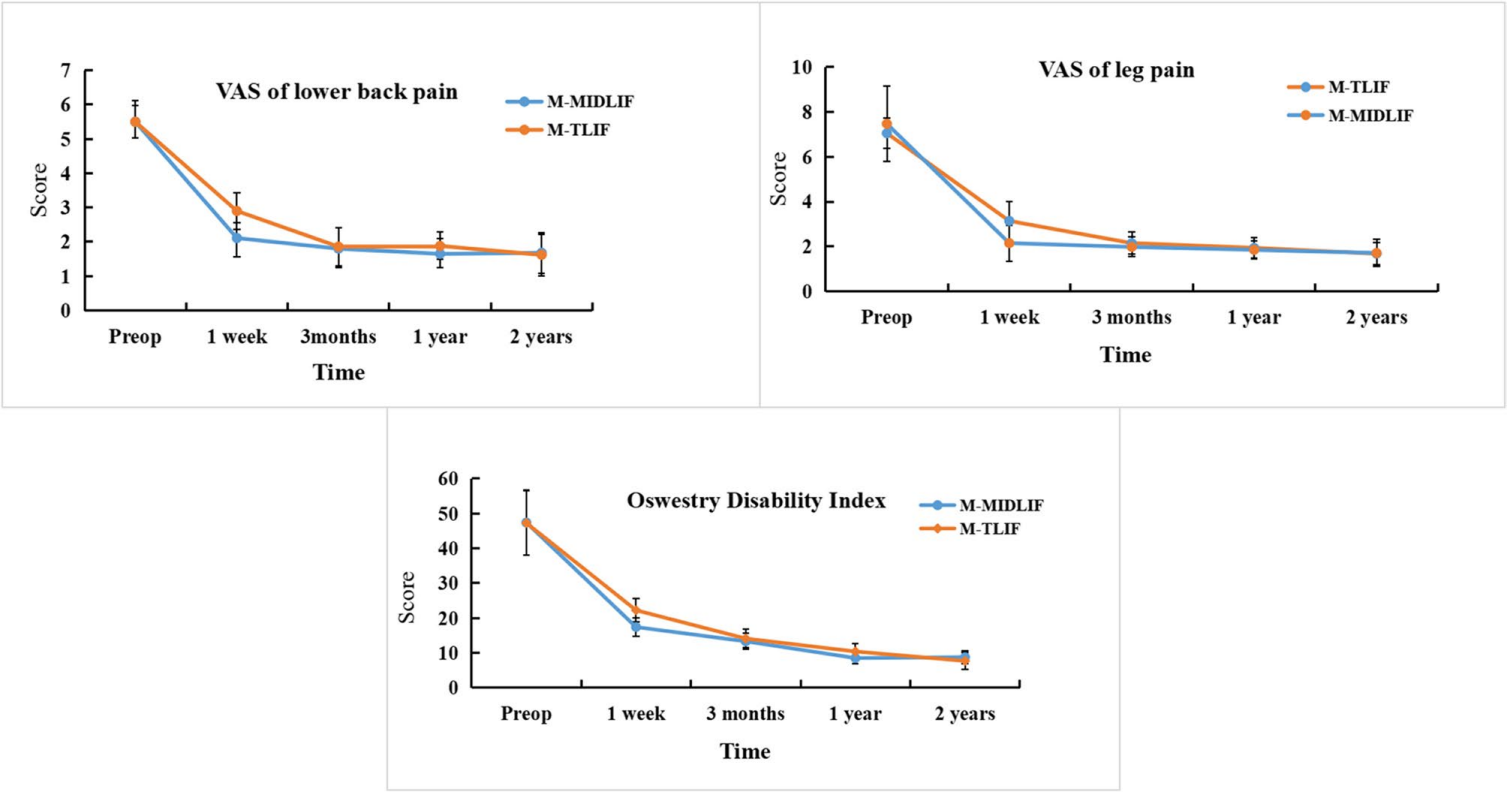
ODI and VAS scores for leg and back pain in the M-MIDLIF and M-TLIF groups

Both groups showed significant improvements in the ODI and VAS scores for low back pain and leg pain at 1 week, 3 months, 1 year, and 2 years postoperatively compared with the preoperative values (*p* < 0.05). In the M-MIDLIF group, the mean VAS score for low back pain decreased from 5.55 ± 0.63 preoperatively to 1.68 ± 0.48 at 2 years postoperatively, whereas in the M-TLIF group, it decreased from 5.50 ± 0.47 to 1.62 ± 0.61 (Fig. 5). Similarly, the mean VAS score for leg pain significantly decreased in both groups, from 7.47 ± 1.68 preoperatively to 1.71 ± 0.61 at 2 years postoperatively in the M-MIDLIF group and from 7.38 ± 1.59 to 1.68 ± 0.48 in the M-TLIF group (Fig. 5). Furthermore, the mean ODI score improved from 47.32 ± 9.42 preoperatively to 8.77 ± 1.87 at 2 years postoperatively in the M-MIDLIF group and from 47.26 ± 9.33 to 7.68 ± 2.46 in the M-TLIF group (Fig. 5). Notably, the M-MIDLIF group demonstrated greater reductions in the ODI and VAS scores for both low back and leg pain at 1 week postoperatively than did the M-TLIF group (*p* < 0.05). However, no statistically significant differences were observed between the two groups at subsequent time points (*p* > 0.05).

### Comparison of radiological data

Among the 56 MCBT screws, 53 (94.64%), 2 (3.57%), and 1 (1.79%) were rated as Grade A, B, and C, respectively. Similarly, for the 56 TASS inserts, 53 (94.64%) and 3 (5.36%) screws were rated as Grade A and B, respectively. Among the 112 screws in the M-TLIF group, 98 (87.5%), 13 (11.61%), and 1 (0.89%) screws were rated as Grade A, B, and C, respectively. The optimal screw position, number of clinically acceptable screws and number of misplaced screws among the MCBT, TASS, and PS groups did not differ significantly (*p* > 0.05). The lateral deviation is the most common direction for MCBT screw misplacement (*p =* 0.002*). The inter-observer agreement for facet fusion grading demonstrated good reliability, with a weighted kappa coefficient of 0.739 (95% CI: 0.541–0.937). The interbody (92.85% vs. 92.85%) and facet joint (83.92% vs. 92.85%) fusion rates did not differ significantly between the two groups. Additionally, the MCBT screws presented a significantly lower FJV rate (3.57%) than the TPS screws (14.29%, *p* 0.047; Table 3).

**Table 3:** Assessment of pedicle screw placement accuracy and proximal facet joint violations of CBT, TASS, and PS

Parameters	M-MIDLIF		M-TLIF		
	Screws		PS	t/Z/X^2^	*p *value
	MCBTS	TASS			
Screw grade					
A	53(94.64%)	53(94.64%)	98(87.50%)	3.514	0.173
B	2(3.57%)	3(5.36%)	13(11.61%)	3.987	0.136
C	1(1.79%)	0	1（0.89%）		1.000*
D	0	0	0	-	-
E	0	0	0	-	-
A+B	55(98.21%)	56(100%)	111(99.11%)		1.000*
C+D+E	1(1.79%)	0	1(0.47%)		1.000*
Direction of screw deviation					
Superior	0	0	0	-	-
Inferior	0	0	0	-	-
Medial	0	3(100%)	13(100%)	12.520	0.002*
Lateral	3(100%)	0	0	12.520	0.002*
FJV grade					
0	54(96.43%)		48(85.71%)	3.953	0.047
1	2(3.57%)		8(14.29%)	3.953	0.047
2	0		0	-	-
3	0		0	-	-
Fusion rate					
Interbody fusion rate	26(92.85%)		26(92.85%)		1.000*
Facet fusion rate	47(83.92%)		52(92.85%)	2.176	0.140

*p-value was calculated using Fisher's precision probability test

## Discussion

TLIF is a minimally invasive lumbar spine surgical procedure that involves a bilateral incision through the Wiltse approach [1, 2]. It has several advantages, such as minimal blood loss, few complications, and maintenance of the PLC [3, 4]. Decompression, fixation, and fusion should be completed within the bilateral incisions. MIDLIF offers minimally invasive benefits by allowing decompression, fusion, and CBT screw fixation in a posterior midline incision [15]. However, CBT screws not only lack easily identifiable and reproducible anatomical landmark indications but also cause damage to PLC to meet placement standards [13, 15, 16]. Limited bone decompression and avoidance of PLC resection can maintain spinal integrity while minimizing tissue damage and reducing the incidence of postoperative instability and postoperative complications [5, 6, 24–26]. Therefore, completing decompression, fixation, and fusion in MIDLIF without PLC impairment has become a new technical challenge.

### Surgical anatomy and the MCBT technique

CBT screws select the facet joint and isthmus as anatomical reference landmarks [11, 13, 14, 27, 28]. However, during the operation, confirming the CBT screw entry point requires excessive exposure of the facet joint and increases the fluorescence examination time. Facet joint degeneration may increase the possibility of nerve and vessel injuries during surgery. The inverted V-shaped crest serves as a well-defined and identifiable landmark for traditional TPS insertion [20]. Therefore, our study proposed a precise anatomical landmark of the internal inverted V-shaped crest to guide MCBT screw placement. Preoperative CT three-dimensional (3D) internal inverted V-shaped crest reconstruction yielded radiographic and intraoperative visual recognition rates of 90.20% and 87.25%, respectively. The internal inverted V-shaped crest is an easily identifiable and reproducible visual landmark that can guide freehand MCBT screw placement. The main challenge in identifying the internal inverted V-shaped crest is its absence or blurriness and facet joint degeneration, which cause its own occlusion. In some patients (9.80%) during surgery, obstruction of the posterior ligament complex makes it difficult to insert MCBT screws, so it is necessary to destroy the posterior ligament complex to ensure accurate insertion of MCBT screws. In MIDLIF, the use of CBT screws to fix the caudal vertebral body requires a surgical incision to be exposed until the vertebral isthmus is reached. TASS fixation is adopted at the caudal vertebral body in M-MIDLIF to shorten the incision length. To preserve the facet joint comprehensively, the insertion point of TASS on the decompressed side might be influenced toward the established insertion point’s inner side during the operation. After partial removal of the inferior articular process, screws can be inserted after the nerve root and pedicle of the vertebral arch are directly visualized. Excellent visualization of TASS entry points results in increased accuracy of TASS within the pedicle [19]. The increasing bone mineral density of facet joints with degeneration effectively contributes to the stability of TASS fixation [19, 29]. Facet joint degeneration and variations in anatomical orientation may complicate the identification of entry points for TASS, thereby increasing the risk of neurovascular injury and TASS misplacement [19].

### Implications of radiological findings

Compared with TPS, CBT screws have entry points located farther from the facet joints, thereby reducing FJV rates, ASD, and muscle atrophy. Proximal facet joint violation not only accelerates facet joint degeneration and adjacent segment disease but also limits the range of lumbar spine motion and increases the risk of ASD [30, 31]. Previous studies have shown that MIDLIF is associated with a significantly lower incidence of adjacent segment disease than TLIF, indicating its superior advantages in improving long-term patient outcomes [32]. In this study, the M-MIDLIF group presented lower FJV rates than the M-TLIF group, and neither group developed symptomatic adjacent segment disease during the study period. However, extended follow-up is still needed to confirm the long-term clinical outcomes. Intraoperative reduction of facet joint destruction influences postoperative lumbar stability [5, 6]. Facet fusion technology has several advantages, such as minimal invasiveness, fewer technology-related complications, and an enhanced single-level fusion rate, which contributes to the postoperative stability of the lumbar spine [7–9]. Facet joint fusion combined with interbody fusion enhances the postoperative stability of the lumbar structure. If the cortical bone of the articular surface is removed extensively for facet fusion, there is an increased risk of facet joint fracture and TASS loosening. Fusion rates between MIDLIF and TLIF have both been similar among these techniques [33]. Several factors affect interbody fusion, such as age, weight, and fixation, as well as the shape, size, material, and endplate position of the cage [34]. In the early stages of lumbar interbody fusion with internal fixation, the internal fixation construct plays a significant role in maintaining lumbar stability. A stable fixation construct facilitates interbody fusion and early functional rehabilitation exercises. Some studies indicate that while CBT screws can increase the screw pullout strength, their resistance to lateral bending and axial rotation is weaker than that of TPS [10–12]. The lateral angle of the MCBT screw was similar to that of the CBT screw, but the cephalad angle was smaller than the CBT screw angle [14, 15]. CBT screws in the lower lumbar vertebrae are difficult to insert along the ideal trajectory even after partial removal of the spinous process; however, their size is smaller than the ideal trajectory of the CBT screw [16, 35]. The ideal screw size for CBT involves a diameter exceeding 5.5 mm and a length greater than 35 mm, with placement extending sufficiently deep into the vertebral body [35]. The most commonly used MCBT screw size is 5.0-5.5 mm in diameter with a length of 30–35 mm. Thus, reducing the cephalad angle of the MCBT screw and changing the entry points favor the selection of longer screws, thereby increasing the cortical bone attachment and the screw-holding force. While this study demonstrated favorable clinical and radiographic outcomes supporting the clinical stability of the MCBT-TASS construct for achieving fusion, we acknowledge the lack of direct biomechanical testing in the current work. Importantly, however, the trajectory design of MCBT screws shares core biomechanical principles with those of CBT screws, which have been extensively studied and shown to significantly enhance screw purchase by maximizing cortical bone contact and improving pullout strength compared with conventional TPS [10–12]. Furthermore, the dimensions and insertion trajectory of TASS, as employed in our technique and referenced from Terai et al., are designed to engage robust bony structures [18, 19]. Crucially, the high interbody fusion rates and facet fusion rates observed in both groups serve as the most compelling indirect clinical evidence for the sufficiency of stability provided by the MCBT-TASS construct during the fusion period. A successful bony union inherently requires a stable mechanical environment. Even if TASS have dimensions similar to TPS [18, 19], their distinct screw trajectory necessitates validation of their biomechanical properties through biomechanical testing. M-MIDLF preserves the facet and the PLC, which also contributes to the postoperative stability of the lumbar spine. After facet fusion and interbody fusion achieve bony fusion, a three-column bony structure is formed, which helps maintain the long-term stability of the lumbar spine. The ability of MCBT screws, in combination with TASS, to provide sufficient stability for interbody fusion might be an important influencing factor.

### Learning curve and clinical outcomes

M-MIDLIF, as a minimally invasive lumbar interbody fusion technique, offers advantages such as reduced surgical trauma. However, this study has several potential limitations. The relatively narrow surgical field may require excessive retraction of muscle tissue to achieve sufficient visualization, potentially compromising surgical precision and maneuverability, increasing the risk of neurovascular injury, and demanding a longer learning curve. The dimensions of the MCBT screws, including their diameter and length, were determined preoperatively via 3D CT scans to assess anatomical parameters such as the width and trajectory of the pedicle. Spine surgeons are highly familiar with the midline approach to the lumbar spine. The primary factors influencing the learning curve of M-MIDLIF are the intraoperative identification of the internal inverted V-shaped crest and the placement of MCBT screws. Preoperative identification of the internal inverted V-shaped crest on 3D CT reconstructions of the lumbar spine can significantly aid in its intraoperative recognition. When the intraoperative identification of the internal inverted V-shaped crest is difficult, reliance on intraoperative fluoroscopic correction or other anatomical landmarks becomes necessary [11, 13, 14, 27, 28]. Although 3D printing, navigation technology, and robot-assisted screw placement can increase the accuracy of screw insertion, these technologies increase patient treatment costs [36–39]. The surgical instruments required for M-MIDLIF are identical to those used in M-TLIF, reducing the time needed for surgeons to familiarize themselves with the instruments. It is recommended that navigation technology be incorporated during the initial phase of training to shorten the adaptation cycle. While the surgical technique described in Terai et al.‘s study may facilitate early adoption of CBT-TASS fixation, the use of the pedicle stump as the entry point for CBT screws not only compromises screw stability and increases the risk of pedicle breach but also disrupts the PLC [19].

In PLIF cases, CBT-TASS fixation offers short-term advantages over Wiltse-approach TPS fixation, including reduced operative time, blood loss, hospital stay, and muscle injury [19]. No significant differences were observed in long-term outcomes, such as spondylolisthesis grade, lordotic angle, fusion rate, or clinical score. Through refinements in surgical technique, the M-MIDLIF group demonstrated significant improvements in intraoperative blood loss, postoperative pain, and functional recovery. M-MIDLIF effectively reduces blood loss, thereby decreasing transfusion requirements in high-risk patients. A reduction in drainage volume not only facilitates a shorter duration of postoperative drain retention but also lowers the risk of postoperative hematoma formation and wound oozing, consequently reducing the probability of infection [40]. Furthermore, M-MIDLIF significantly minimizes the extent of dissection in the lumbar paraspinal muscles and effectively preserves the integrity of the PLC during surgery. This approach helps preserve lumbar spinal stability while reducing the incidence of postoperative muscle atrophy and chronic back pain [10–12, 41–43]. Reduced surgical dissection also alleviates postoperative wound pain, enabling earlier ambulation to prevent postoperative complications [33]. Concurrently, this approach supports the timely initiation of functional exercises, accelerating the overall rehabilitation process [44, 45]. Collectively, these advancements comprehensively enhance perioperative safety and optimize long-term functional recovery outcomes.

### Limitations

First, this study excluded patients with multi-segment lumbar degenerative disease, revision surgery, or severe osteoporosis. While this approach effectively controlled for confounding variables and ensured intergroup comparability, it substantially limited the generalizability of the research findings to real-world clinical settings. Future investigations should incorporate stratified analyses of bone density subgroups, conduct multicenter validation of safety profiles in complex cases, and employ biomechanical modeling to optimize screw system configurations. Second, the absence of biomechanical validation for MCBT-TASS constructs, including pullout resistance and fatigue strength through in vitro experiments, also weakens the theoretical foundation of this study. Third, despite propensity score matching, subgroup analyses of complications and specific radiological parameters may lack the statistical power to detect subtle differences due to sample size constraints. Finally, although M-MIDLIF has demonstrated perioperative advantages in terms of minimal invasiveness, future studies adopting prospective multicenter designs, extended follow-ups, and interdisciplinary approaches (e.g., finite element modeling and dynamic imaging techniques) are necessary for further validation.

## Conclusion

M-MIDLIF can achieve decompression, fixation, and fusion via a median incision while preserving the integrity of the posterior ligamentous complex. In the treatment of single-level lumbar degenerative disease, M-MIDLIF has comparatively enhanced minimally invasive advantages over M-TLIF during the perioperative period while maintaining non-inferior clinical safety and efficacy relative to M-TLIF.

## Data Availability

No datasets were generated or analysed during the current study.
